# Relation of Physicians to the Public—Medical Jurisprudence*Read before the Brazos Valley Medical Association at Marlin, Texas, May 9 and 10, 1899.

**Published:** 1899-06

**Authors:** R. L. Henry

**Affiliations:** Waco, Texas


For Texas Medical Journal, the Official Organ of the Brazos Valley Medical Association.

### *Relation of Physicians to the Public—Medical Jurisprudence.

BY HON. R. L. HENRY, M. C., WACO, TEXAS.

*Mr. President and Gentlemen of the Brazos Valley Medical Association:*

The honor implied by your courteous request to address this Association is duly appreciated by me. Having accepted your generous invitation, I shall undertake to discuss a few practical propositions which should certainly interest those striving to reach the true relation of the physician and surgeon to the public. It has long been impressed upon me that the medical fraternity is not properly educated in certain branches of the law, and the legal fraternity possesses a frightfully small *quantum* of medical knowledge. Whether the term "Medical Jurisprudence" be a misnomer or not, it now has an accepted and fixed meaning. A noted standard author has defined it to be "that science which teaches the application of every branch of medical knowledge to the purposes of the law; hence its limits are, on the one hand, the requirements of the law, and, on the other, the whole range of medicine." It has frequently occurred to me in the practice of my profession, that the law schools have most woefully neglected an important study in not requiring on the part of the students a thorough and exhaustive study of some standard work on Medical Jurisprudence. No law course is complete without it. It often happens that attorneys betray painful ignorance of important questions of medical jurisprudence arising in a case on trial. And the lawyer who is properly informed on the subject is frequently the complete master over his adversary. I deliberately state that in these modern days no lawyer is completely qualified to go into the court house and correctly try many criminal and civil cases until he has thoroughly grounded himself in medical jurisprudence. On the other hand, it is incumbent upon the physician who loves his profession and cherishes justice, to know something of the fundamental rules of law and evidence. Often the correct solution of the most important points in a criminal or civil case will depend upon the settlement of some question of medical jurisprudence by the statement

*Read before the Brazos Valley Medical Association at Marlin, Texas, May 9 and 10, 1899.

of a member of the medical fraternity. His word frequently is absolutely the turning point. Then, how important it is that he should know precisely the verity of his statements and that his evidence should be given with scrupulous impartiality. There is a sacred reciprocal duty existing between the public and the enlightened physician and surgeon. The public should protect him in the practice of his profession against frauds and charlatans; and he should bring to the demands of the public the ripest fruits of an honorable and unbiased knowledge of the truths of the great questions to which he is devoting his life. When he enters the witness stand he should come without favor or partiality, bringing at all times the best knowledge of his profession, ever striving to vindicate the learning, skill and integrity thereof. No medical man can tell when he may be called from his supposed obscurity to testify concerning some momentous question affecting the public. His highest ambition should, therefore, be to fairly settle such question by a profound knowledge and complete elucidation of the point at issue. Hence I contend that the medical man has not finished his education until he has thoroughly studied some standard work on evidence and has to some degree mastered the general rules and fundamental propositions of law to which medical jurisprudence generally pertains. Let him carefully study Greenleaf on Evidence, or some other first class authority. In short, you should inform yourselves on medico-legal questions now daily arising, or you cannot be a well rounded member of your profession. I know from experience to what extent my profession must implicitly rely upon yours for the correct determination of these questions. I say, without intending to offensively criticise, that both professions are woefully remiss in their study of these questions. Only a few days since, I read of two brethren of our respective professions being thrown under the tender protection of one another. A young quack doctor was charged in the courts with producing a criminal abortion. A young lawyer defended him, and based his defense on that section of the statute of frauds in regard to parol agreements, which provides that "no person shall be held to answer for the debt, default or *miscarriage* of another, unless the contract be in writing;" and as his client did not agree in writing to answer for the *miscarriage* he was not amenable to the law. You may be sure that the educated lawyer who takes any pride in his profession will carefully prepare and fortify himself upon all questions of medical jurisprudence pertaining to his case; and the medical man who properly conceives his duty should completely master all points of legal medicine involved before he goes upon the witness stand, so that he may creditably undergo the severest cross-examination. The skilled and ambitious lawyer will test him to the uttermost limits of his knowledge on the issues involved.

The expert medical man is frequently called into court, and, it seems to me, without just compensation. In this State both expert and inexpert witnesses are placed upon the same footing under the law with reference to compensation. The State of Texas in criminal prosecutions pays no witness fees except to attached witnesses carried from one country to another. And then all witnesses are placed in the same attitude in regard to costs. No costs or fees can be taxed or paid except those provided for by express statute, and no provision has been made by the State except for attached witnesses. This is a serious question to the expert who is dragged away from his professional business, and should be here thoroughly understood. The question has come before our Supreme Court once. It was in the case of Fears vs. Nacogdoches County. A practicing physician, upon summons, made post mortem examinations upon two dead bodies. He made the examinations because he was threatened with attachments by the justice. He presented his claim for forty dollars to the Commissioners' Court and they allowed him ten dollars. He sued the county. He obtained judgment for ten dollars, and then appealed to the Supreme Court. That court distinctly held that under our statutes there is no provision for the compensation of a physician summoned to conduct a post-mortem examination in an inquest. Chief Justice Gaines said: "A post-mortem examination at a coroner's inquest is frequently necessary for the detection and punishment of crime. It does not seem just to impose this duty without compensation upon a learned and enlightened profession whose custom it is not to refuse the calls of charity. But they must look to the Legislature for relief. We can only declare the law as we find it; and as it now stands we think there is no provision for their compensation." This broad suggestion thrown out by the learned Chief Justice has gone unheeded by the Legislature. It seems to me that his suggestions are pertinent and eminently just. And thus Texas laws now stand. In many States statutes have been passed providing just compensation for the attendance of expert witnesses, notably Iowa, North Carolina, Rhode Island and Minnesota. Of course, when such a witness testifies to ordinary facts falling under his notice he stands in the attitude of other witnesses.

In 1875 a very important decision on this question was rendered in the case of Ex parte Dement in Alabama. The opinion was learned and exhaustive, and the court held, that a physician, like any other person, may be called to testify as an expert in a judicial investigation, whether it be civil or criminal in its nature, without being paid for his testimony as for a professional opinion; and that upon refusal to testify he may be punished for contempt. This opinion has been followed in many States. It seems to me, after a careful perusal of the law and decisions, the weight of authority is with the Dement case from Alabama, and that the only safe course is for the expert witness to testify if ordered so to do by the court and not demand extra compensation. He owes a duty to the public. The right to compel him to give these services is an incident to the exercise of government itself, just as the juror and soldier are obliged to serve their country. After all it rests upon the maxim *salus populi suprema lex.* The reciprocal duty of the government is to protect the medical man in the dignity and efficiency of his profession against quacks and empirics. It appears clear that the State can "impound" your services without paying extra compensation to you as expert witnesses. The Legislature should remedy the evil. Some equitable relief should be speedily given by that body. It would be a matter of simple justice to a learned, noble and unselfish profession. A few lines by the Legislature can accord this just and deserved right to your profession in pursuance of the strong language of our eminent Chief Justice.

I wish to call your attention to another point about which is discoverable much lack of knowledge, both in your profession and mine. Frequently I have heard distinguished medical men and lawyers proclaim that the sanctity of privileged communications from patients to the medical fraternity pertained the same to them as to the legal profession. If any proposition of law is well established it is the negative of this question. At common law the medical man has no privilege to avoid giving evidence about a matter communicated to him by his patient. This is still the law in most States except where statutory provisions have abrogated it. No matter how sacred, private or confidential the statement may be, when the medical man is put upon the witness stand he must disclose every such communication without reserve. The lawyer and medical man in this relation stand upon an entirely different footing, although the relations are certainly equally confidential and sacred. The former is protected and can claim his privilege. The latter cannot. This is undoubtedly an unjust deformity of the law to a great extent. I have come to this conclusion after much meditation and study on this point. And the fact that Legislatures have been slow to act is proof positive that it is a nice and doubtful question. There is no favoritism here intended to the legal profession. There is no intention of an unjust discrimination. The two professions here are upon different bases. The lawyer is an officer of the court and an integral part of its functions. He is licensed by it and his professional conduct measured and regulated entirely by the courts, and to them he is strictly accountable as an arm of the judicial machinery. The medical man is not. He is only accountable to the public and to his profession for his professional acts. The reason for this distinction is a due regard for the administration of justice. The business of the courts could not proceed without this apparent distinction, for no man would entrust his case to the lawyer unless he were so shielded by the doctrine of privileged communication from client to attorney. Of course no medical man should reveal such communications until required to do so by the court, and then his conduct would not be reprehensible, but perfectly justifiable. This incident is related by an eminent authority on Medical Jurisprudence: On cross-examination a medical witness was asked, in a case of murder by poison, what remedy or antidote he had employed when he first reached the deceased. He appealed to the trial judge to know if he must answer the question. The judge replied: "Yes, unless you have reason to believe that your antidote killed the deceased. In that case you are not bound to answer it." It is hardly necessary to state that the witness answered the query with startling alacrity. This instance clearly reveals the settled doctrine of the law on this point. It is thoroughly determined that the medical witness must answer every interrogatory which does not incriminate himself. Long since, in the case of the Duchess of Kingston, this point became established law, and has been uniformly followed. We are then brought to this just conclusion, that the medical man should be reuired to disclose any communication from his patient, no matter how confidential, where the due and proper administration of public justice requires it. Beyond this he should not be required to go, and the Legislature should speedily accord this protection to him by passing a statute covering the point. Some States have already taken action in that direction. In such States the physician or surgeon is either forbidden or not obliged to reveal secrets communicated to him by his patient. The statutes exist in Arkansas, California, Indiana, Iowa, Michigan, Minnesota, Missouri, Montana, New York, Ohio and Wisconsin. The seal upon the physician's lips is not removed by death of the patient. The seal of secrecy should be kept there, except where the communication is unlawful and against public policy and morality. When we have provided for the due and proper administration of public justice we have gone far enough, and then the medical profession should be jealously protected in their private and sacred relations to their patients. Let Texas follow in the wake of the States above mentioned. As the law now stands in Texas a physician or surgeon, either in a civil or criminal case, except where his testimony would incriminate himself, must lay bare in court every secret taken from the sacred precincts of his patient's home.

Not only should the medical expert be treated with reasonable consideration as regards compensation and the sanctity of his medical secrets, but the State should aid the profession to guard against impostors, quacks and charlatans. The Constitution of 1876 contains this language "The Legislature may pass laws prescribing the qualifications of practitioners of medicine in this State and to punish persons for malpractice, but no preference shall ever be given to any schools of medicine." Constitution, Article 16, Section 31. In pursuance of this constitutional provision the Texas statutes provide for the appointment of boards of medical examiners by the various district judges. Then it is made a grave misdemeanor to practice medicine without obtaining the proper certificate of qualification from the duly authorized board of medical examiners, or without having a diploma from some accredited medical college, chartered by the Legislature of the State, or its authority, in which the same is situated. And then a failure to file the certificate or diploma with the clerk of the district court of the county in which the physician resides is denounced as a crime. Article 398, Penal Code. These laws have been passed for the protection of reputable medical practitioners and for the benefit of society against frauds and quacks. We have a right to demand the most diligent scrutiny against such impostors, and wholesome statutes to that end cannot be too severe.

The test of the medical board for admitting medical practitioners in this State should be much more rigid than the qualifications for admitting attorneys. For the attorney is under the constant control and watchful eye of the courts and can be disbarred and expelled at any time. But the medical practitioner once admitted by the board is permitted to deal with the health and lives of the people without let or hindrance. I would not have the laws militate against any particular school of medicine, but let the medical fraternity see to it that the very highest class of boards be appointed and that the tests for admission through them be stringent, just and effective. In this way only can they protect themselves against frauds and acts of empiricism, and shield society from the miserable impostor and charlatan that should be so heralded to all the world.

In the case of Kennedy vs. Schultz, Mr. Justice Fly of the Court of Civil Appeals, held that Kennedy, who was suing Schultz for $1,200 for services rendered as a physician, could not recover the sum because he had failed to procure a certificate from the medical board, and because such certificate was not duly recorded with the district clerk of Bexar county. The learned justice held the statute mandatory, and should be commended for taking this honorable and vigorous step in behalf of your profession. The court held that the statute was not in violation of the constitution, but in pursuance thereof. The court held that any contract made in defiance of the statutory provisions for a medical board and examinations thereunder was against public policy, and could not be enforced. Again, in the case of Muth vs. San Antonio Street Railway, similar points were brought before the same court. Mr. Justice Neill delivered an able opinion, in which he held that although Dr. Kennedy held a diploma from a reputable medical college in the United States he was not entitled to recover for medical services for attendance upon Muth's daughter without showing that he had obtained an appropriate certificate of qualification from the proper medical board, and that the same had been filed with the district clerk. Muth was undertaking to recover from the street railway company fees claimed to have been paid Kennedy for attending upon his injured daughter. But the court held that Kennedy's services were illegal and in violation of the statute, in that he had not procured the proper certificate from the appropriate medical board, and being in violation of law, neither Kennedy nor Muth could recover the amounts alleged to be due. These decisions are along the right lines, and should be promptly reinforced by adequate legislation that will as completely as possible purge the medical profession of all frauds and quacks, and protect society against them. It seems to me that we cannot be too prudent about this matter, and the time is now ripe for your fraternity to supplement these statutes and decisions and perfect the laws. When these matters are discussed before the public, I am convinced that all reputable attorneys and good citizens will aid you in purifying your profession. When the legal fraternity are brought into closer relation with their medical brethren, and we both come to better understand and appreciate the great importance of legal medicine, by our joint efforts more wholesome and effective laws may be secured pertaining to our rights as noble professions. It seems to me that to a great extent the medical profession does not sufficiently acknowledge the importance of medical jurisprudence. There is a very grave responsibility resting upon expert witnesses from your profession in the trial of important cases. Most frequently the jurors, the lawyers and the court are helplessly dependent upon you. Then think how important it is that you thoroughly understand the question and return an accurate, unbiased and correct answer. If we would arrive at the correct solution of an issue we must rely upon your moral and intellectual honesty and conduct when you are called as experts. Your answers should be in perspicuous and accurate language without pedantry. I cannot better illustrate the necessity of this course than by restating an anecdote noticed in a standard medical authority. It may be ancient, but it beautifully illustrates the helplessness of my brethren when you are not kind enough to give us an answer in the plainest English. On a trial for an assault, which took place some years since at the Assizes, a surgeon, in giving his evidence, informed the court that in examining the prosecutor he found him suffering from a severe contusion of the integuments under the left orbit, with great extravasation of blood and ecchymosis in the surrounding cellular tissue, which was in a tumefied state. There was also considerable abrasion of the cuticle. The judge asked: "You mean, I suppose, that the man had a black eye?" The witness replied, "Yes." The judge—"Then, why not say so at once?"

When the medical expert goes upon the witness stand it is then that the great importance and influence of his relation towards society and the public are vividly revealed. If he is the complete master of his profession all questions may be correctly determined. If he be a shrewd impostor, morally and intellectually dishonest, most grievous wrongs will be done the public and private individuals. For these reasons the polite, deliberate, clean and masterly physician and surgeon will ever be greatly honored in the hearts of his countrymen and the forums of justice.

